# Nullbasic, a Potent Anti-HIV Tat Mutant, Induces CRM1-Dependent Disruption of HIV Rev Trafficking

**DOI:** 10.1371/journal.pone.0051466

**Published:** 2012-12-10

**Authors:** Min-Hsuan Lin, Haran Sivakumaran, Ann Apolloni, Ting Wei, David A. Jans, David Harrich

**Affiliations:** 1 Queensland Institute of Medical Research, Molecular Virology Laboratory, Herston, Brisbane, Australia; 2 School of Chemistry and Molecular Biosciences, University of Queensland, St. Lucia, Queensland, Australia; 3 Department of Biochemistry and Molecular Biology, Monash University, Clayton, Victoria, Australia; Centro de Biología Molecular Severo Ochoa (CSIC-UAM), Spain

## Abstract

Nullbasic, a mutant of the HIV-1 Tat protein, has anti-HIV-1 activity through mechanisms that include inhibition of Rev function and redistribution of the HIV-1 Rev protein from the nucleolus to the nucleoplasm and cytoplasm. Here we investigate the mechanism of this effect for the first time, establishing that redistribution of Rev by Nullbasic is not due to direct interaction between the two proteins. Rather, Nullbasic affects subcellular localization of cellular proteins that regulate Rev trafficking. In particular, Nullbasic induced redistribution of exportin 1 (CRM1), nucleophosmin (B23) and nucleolin (C23) from the nucleolus to the nucleus when Rev was coexpressed, but never in its absence. Inhibition of the Rev:CRM1 interaction by leptomycin B or a non-interacting RevM10 mutant completely blocked redistribution of Rev by Nullbasic. Finally, Nullbasic did not inhibit importin β- or transportin 1-mediated nuclear import, suggesting that cytoplasmic accumulation of Rev was due to increased export by CRM1. Overall, our data support the conclusion that CRM1-dependent subcellular redistribution of Rev from the nucleolus by Nullbasic is not through general perturbation of either nuclear import or export. Rather, Nullbasic appears to interact with and disrupt specific components of a Rev trafficking complex required for its nucleocytoplasmic shuttling and, in particular, its nucleolar accumulation.

## Introduction

Both the Human immunodeficiency virus type-1 (HIV-1) Tat and Rev proteins are encoded by two exons arranged in alternative reading frames on fully spliced viral mRNA [1]. Tat and Rev are similar in size; Tat is typically 101 amino acids long and Rev is typically 116 amino acids long, and both have RNA binding domains composed of arginine and, in the case of Tat, lysine residues which bind to different HIV-1 RNA stem loop structures. Tat binds to an RNA structure in the 5′ untranslated region (UTR) of all viral transcripts called the Trans-Activation Response element (TAR), while Rev binds to an intronic region retained by incompletely spliced transcripts called the Rev Response Element (RRE). The RNA binding domains of both proteins also function as a nuclear/nucleolar localization signal (NLS/NoLS), although recent evidence implies that Tat may passively enter the nucleus by diffusing through nuclear pores [2]. Both proteins are localized primarily in the nucleus; Tat is observed throughout the nucleoplasm with nucleolar accumulation, whereas the nucleocytoplasmic shuttling Rev concentrates in the nucleolus in addition to localizing to the nucleoplasm and, to a lesser extent, to the cytoplasm.

**Figure 1:**
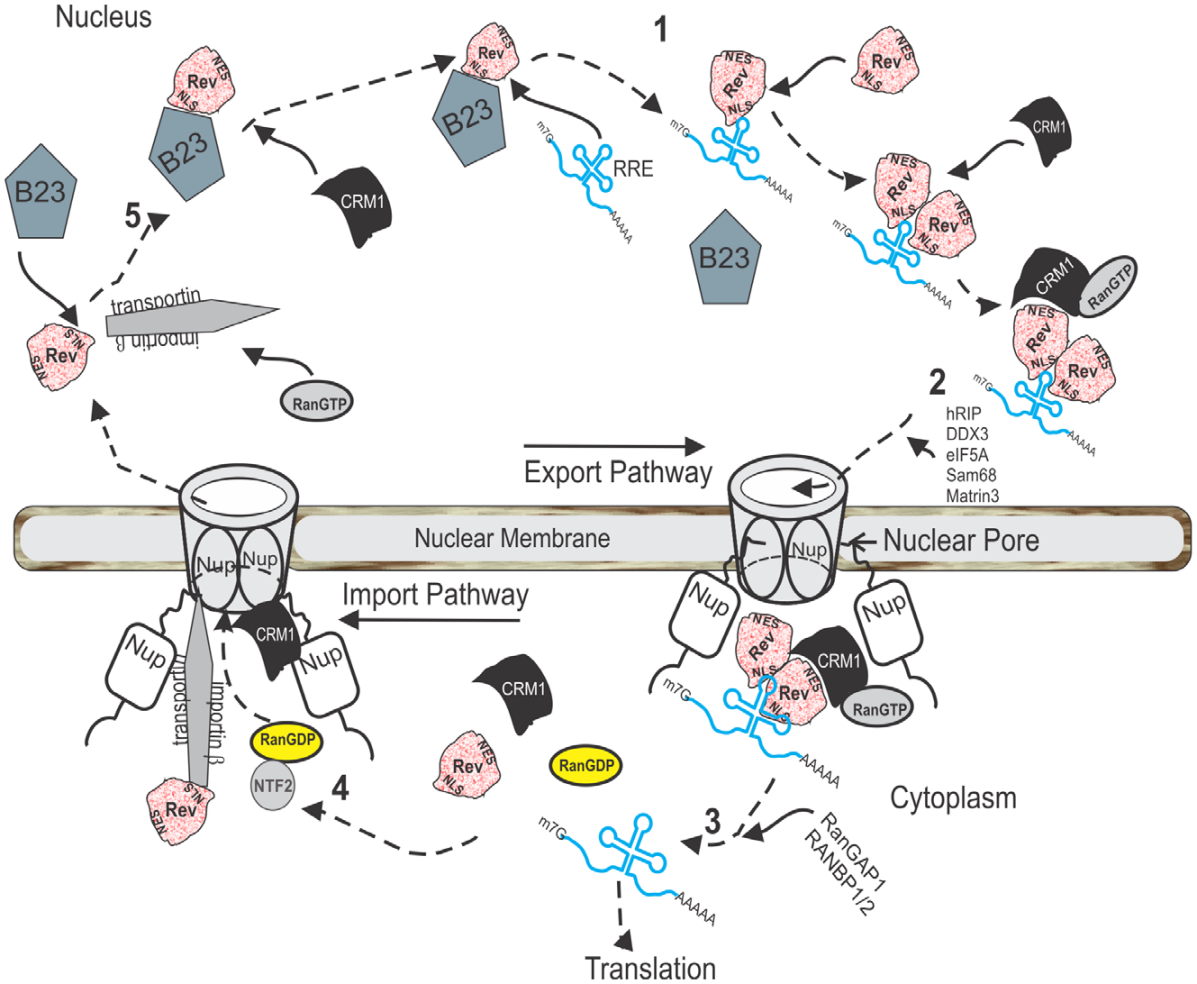
The nucleocytoplasmic trafficking of Rev. Summary of the current understanding of molecular events regulating Rev trafficking within the infected cell [3], [4].

Trafficking of Rev in cells has been studied extensively (Fig. 1) [3], [4]. In the nucleolus, Rev promotes the nuclear export of various HIV-1 mRNAs by directly binding to singly-spliced and unspliced viral transcripts via the RRE contained therein (Fig. 1, step 1). Exportin 1 (also called CRM1 and XPO1) binds to Rev through a nuclear export signal (NES; HIV-1_NL4-3_ Rev amino acids 73 to 84, LQLPPLERLTLD) [5], [6], [7], which leads to colocalization of Rev and CRM1 in the nucleolus and subsequent export of the Rev:mRNA complex from the nucleus to the cytoplasm (Fig. 1, step 2). Many other cellular proteins can contribute to Rev nuclear export, including hRIP/Rab, eIF5A, DDX3, DDX1, RNA helicase A, and PIMT that act through Rev, and Matrin 3 and Sam68 that bind to viral mRNA [3], [8], [9], [10], [11], [12]. The Rev:mRNA complex disassembles in the cytoplasm (Fig. 1, step 3) allowing Rev to recycle back to the nucleus using the transportin 1 or importin β nuclear import pathways (Fig. 1, step 4) [3]. Once Rev enters the nucleus, nucleophosmin (B23) facilitates transport of Rev to the nucleolus (Fig. 1, step 5) [13]. B23 is reported to be necessary for the nucleolar localization of both Rev and Tat through interaction with their respective basic domains [13], [14], [15], [16], [17].

We recently described a mutant of the two-exon HIV-1 Tat protein, termed Nullbasic, that exhibits antiviral properties by inhibiting multiple steps of the HIV-1 replication cycle [18]. Nullbasic was created by replacing the entire arginine-rich RNA binding domain of wild type Tat with glycine and alanine residues. Like similarly mutated one-exon Tat mutants, Nullbasic exhibits transdominant negative effects on Tat-dependent HIV-1 gene expression [18]. However, unlike previously reported mutants [19], [20], [21], Nullbasic also effectively suppresses the steady state levels of unspliced and singly-spliced viral mRNA, an activity caused by the inhibition of HIV-1 Rev activity [18]. The inhibition by Nullbasic was attributed, in part, to a subcellular redistribution of Rev from the nucleolus to the nucleoplasm and cytoplasm, but precisely how Nullbasic mediates this effect is unknown.

In this study, we investigated the effect of Nullbasic on the nucleocytoplasmic transport machinery responsible for Rev trafficking. Not surprisingly, Rev recruited and colocalized with several cellular proteins in the nucleolus, including CRM1, B23 and nucleolin (C23). However, coexpression of Nullbasic with Rev resulted in the unexpected redistribution of CRM1 and other nucleolar proteins from the nucleolus to the nucleoplasm in a Rev-dependent manner, whereas Nullbasic did not affect the distributions of these nucleolar proteins when expressed alone. Our experiments support the possibility that Nullbasic interferes with an unknown component of the Rev nucleocytoplasmic transport complex required for the nucleolar organization and function of Rev.

## Materials and Methods

### Cell Lines, Transfections and Drug Treatments

HEK293T and HeLa cells were cultured in RPMI 1640 medium supplemented with 10% (v/v) newborn bovine serum (Invitrogen) and 1% (v/v) penicillin-streptomycin. All cells were typically incubated at 37°C in a humidified 5% CO_2_ atmosphere. When 50% –80% confluent, cells were transfected with desired plasmids using FuGENE 6 transfection reagent (Roche Applied Science) according to the manufacturer’s instructions. At 24 h post-transfection, cells were harvested for further analysis. In certain experiments where Rev nuclear export was blocked, Leptomycin B (Sigma-Aldrich) was added to growth medium at a final concentration of 20 nM and incubated for 2 h before cell fixation.

### Plasmid Constructs

The two-exon Tat (101 amino acids) expression plasmid with FLAG epitope (Tat-FLAG) was a gift from Monsef Benkirane, Institute de Génétique Humaine, France. GFP_2_-cNLS and GFP_2_-M9core plasmids were gifts from Ralph Kehlenbach, Georg-August-University of Göttingen, Germany [22]. The GFP_2_ expression plasmid was obtained from Addgene, plasmid #20738. The construction of the Nullbasic-FLAG and Myc-Rev plasmids have been previously described [18]. The pGCH infectious molecular clone is a HIV proviral plasmid which expresses authentic HIV-1 RNA using the CMV immediate early promoter. The plasmid consists of a pGEM7z (-) (Promega) backbone containing the CMV promoter fused so that the transcription initiation site uses the correct HIV-1+1 position. With respect to the HIV-1 transcription start site, HIV-1 sequences +1 to +933 from the HIV-1 pNL4-3 proviral plasmid (obtained from the ADIS Research and Reference Reagent Program, plasmid #114) were inserted into the pGEM7z (-) via *MluI* and *SphI* restriction enzymes sites and +934 to +9251 from the HIV-1 pNL4-3 were inserted into the pGEM7z (-) via *SphI* and *XbaI* restriction enzymes sites. The HIV-eGFP expression plasmid was engineered by inserting an eGFP sequence from pcDNA3.1-eGFP into the HIV-1 *nef* gene in pGCH proviral expression vector through *Bam*HI and *Xba*I restriction enzyme sites. The expression vector encoding Nullbasic-mCherry was generated by polymerase chain reaction (PCR) amplification of mCherry from pmCherry-LacRep (obtained from Addgene, plasmid #18985), which was then inserted into the Nullbasic-FLAG plasmid via *Bsr*GI and *Xho*I restriction enzyme sites.

### Co-immunoprecipitation Assays and Western Blotting

2.5×10^6^ HEK293T cells grown in 10 cm^2^ dishes for 24 h and were transfected with 4 µg of Myc-Rev plasmid along with 2 µg of either Tat-FLAG or Nullbasic-FLAG plasmids. At 24 h post-transfection, the cells were washed twice with PBS and lysed at 4°C for 30 min with lysis buffer (50 mM Tris-HCl, pH 7.4; 150 mM NaCl; 1 mM EDTA; 1% [v/v] Triton X-100; protease inhibitor cocktail [Roche]). Cell lysates were centrifuged at 12,000×g for 10 min and clarified supernatants were collected. FLAG-tagged proteins and interacting factors were captured using anti-FLAG M2 affinity gel (Sigma-Aldrich) and eluted with sample buffer (125 mM Tris-HCl, pH 6.8; 4% [v/v] sodium dodecylsulfate (SDS); 20% [v/v] glycerol; 0.004% [w/v] bromphenol blue). The eluates were then separated by sodium dodecylsulfate – polyacrylamide gel electrophoresis (SDS-PAGE) and electro-blotted onto a polyvinylidene fluoride (PVDF) membrane (Pall) using a semi-dry transfer system (Bio-Rad Laboratories). Tat-FLAG and Nullbasic-FLAG proteins were detected with a rabbit anti-FLAG polyclonal antibody (Cell Signalling Technology). Myc-Rev was detected with a rabbit anti-Myc polyclonal antibody (Cell Signalling Technology). Endogenous CDK9 was detected with a rabbit anti-CDK9 monoclonal antibody (Cell Signalling Technology). Primary antibodies were detected with houseradish peroxidase (HRP)-conjugated goat anti-rabbit antibody (Invitrogen).

### Indirect Immunofluorescence

HeLa cells were grown on glass coverslips and transfected with appropriate expression vectors. After 24 h, the cells were fixed in 3% (w/v) paraformaldehyde at room temperature for 10 min and quenched with 50 mM NH_4_Cl for 5 min. Cells were then permeabilized with 0.1% (v/v) Triton X-100 for 15 min and blocked in 10% (v/v) normal goat serum (Millipore) for 15 min. Cellular endogenous proteins were detected with rabbit anti-CRM1 polyclonal (Santa Cruz Biotechnology), rabbit anti-fibrillarin monoclonal (Cell Signalling Technology), mouse anti-nucleophosmin monoclonal (Invitrogen) and mouse anti-nucleolin monoclonal (Santa Cruz Biotechnology) antibodies, and Myc-Rev was probed with either mouse anti-Myc monoclonal antibody (Millipore) or rabbit anti-Myc polyclonal antibody (Cell Signalling Technology). Rev expressed from proviral HIV-1 was probed with a mouse anti-HIV-1 Rev monoclonal antibody (Santa Cruz Biotechnology). Primary antibodies were detected with FITC-conjugated goat anti-mouse or anti-rabbit antibodies (Invitrogen) and Alexa Fluor 647-conjugated goat anti-rabbit (Invitrogen), Cy5-conjugated goat anti-mouse antibodies (Invitrogen) or Cy3-congugated goat anti-rabbit antibodies (Invitrogen) (to detect the rabbit anti-FLAG polyclonal antibody). Nuclei were stained with 1 µM 4′,6-diamidino-2-phenylindole (DAPI, Invitrogen). Finally, coverslips were mounted onto slides with ProLong Gold antifade reagent (Invitrogen). Fluorescent images were captured using a Leica TCS SP2 confocal scanning microscope (Leica Microsystems) and DeltaVision deconvolution microscope (Applied Precision) with 63× objective lenses and standard lasers and filters for FITC, mCherry, Cy5, Cy3 and DAPI fluorescence.

## Results

### Nullbasic-induced Rev Mislocalization is not due to an Interaction between the Two Proteins

**Figure 2:**
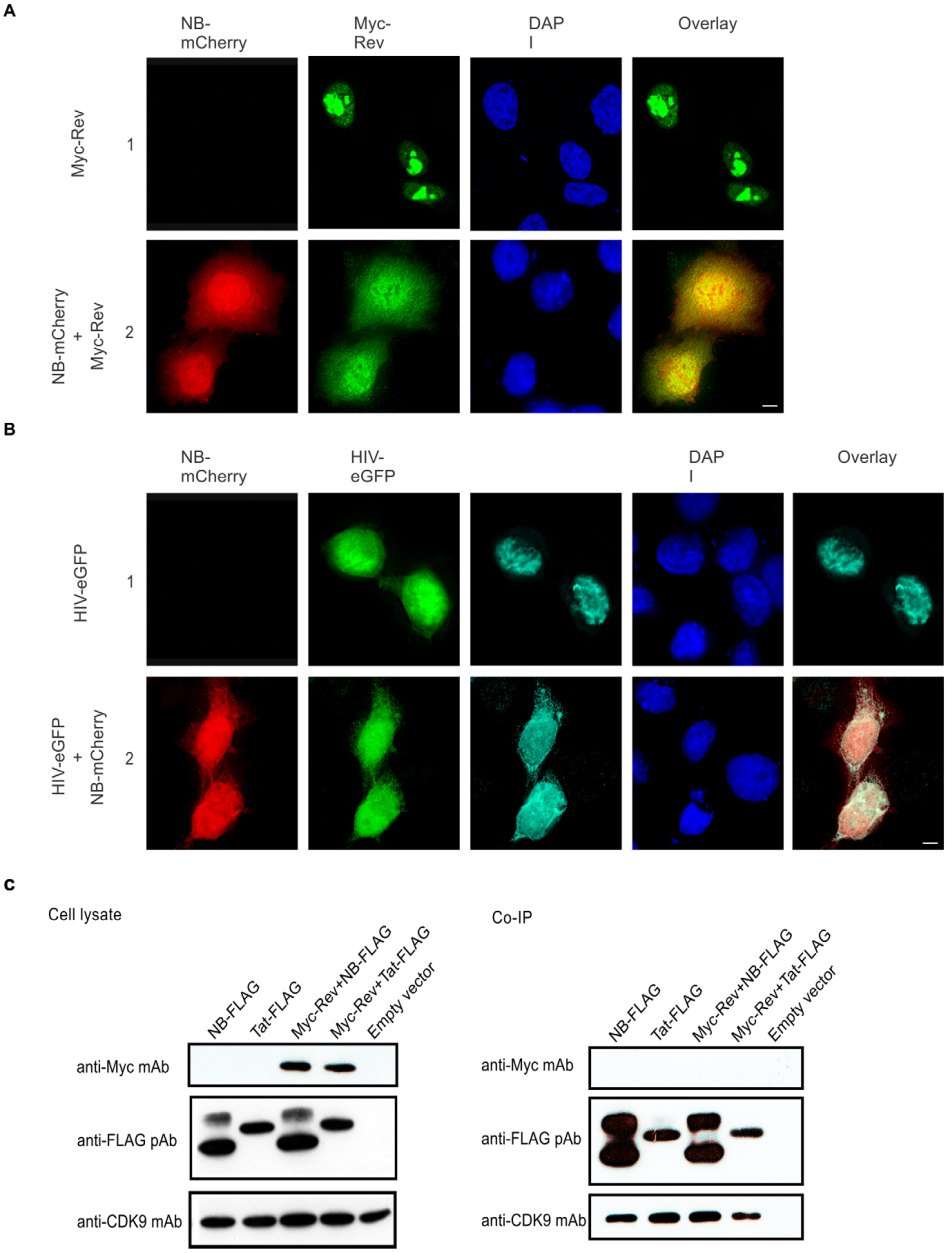
Nullbasic induces Rev redistribution without protein interaction. (A) HeLa cells expressing Myc-Rev alone (row 1) or with Nullbasic (NB)-mCherry (row 2) were fixed and immunostained with anti-Myc antibody before being analyzed by fluorescence microscopy for Myc-Rev (green) and NB-mCherry (red) subcellular localizations. Nuclei were stained with DAPI. The overlay panels show the merge of the Myc-Rev and NB-mCherry panels. The figure is representative of four fields each from four independent experiments. (B) HeLa cells were transfected to express HIV-eGFP alone (row 1) or with NB-mCherry (row 2). Fixed cells were immunostained with anti-Rev antibody before the subcellular localizations of Rev (cyan) and NB-mCherry (red) were visualized by fluorescence microscopy. Expression of HIV-eGFP was confirmed in the FITC channel (green). Nuclei were stained with DAPI. The overlay panels show the merge of the Nullbasic-mCherry and Rev panels. Figures are representative of five fields each from four independent experiments. (C) HEK293T cells were transfected with either empty vector (pcDNA3.1) or plasmids expressing Nullbasic (NB)-FLAG alone, Tat-FLAG alone, or Myc-Rev with either NB-FLAG or Tat-FLAG, as indicated. The FLAG-tagged proteins and their interacting factors were immunoprecipitated using anti-FLAG beads. Total cell lysates (left) and immunoprecipitated proteins (right) were subjected to western blot analysis using anti-FLAG and anti-Myc antibodies. The endogenous protein CDK9, detected using anti-CDK9 antibody, was used as a positive control for Tat interaction. The figure is representative of four independent experiments. The white bar in last panel is equal to 10 µm.

We previously observed that Nullbasic induces considerable redistribution of Rev within the cell, from the nucleolus to the nucleoplasm and cytoplasm, leading to significant down regulation of steady state levels of unspliced or singly-spliced viral mRNA [18]. To investigate whether Nullbasic is able to alter Rev’s subcellular localization in the context of HIV-1, we visualized the subcellular distribution of Rev derived from HIV-1 provirus in the presence or absence of Nullbasic. To overcome transcriptional inhibition caused by Nullbasic [18], we utilized a CMV-driven HIV-1 proviral plasmid, pGCH, which expresses viral factors independent of Tat. The plasmid also has the eGFP gene inserted into the *nef* reading frame (here called HIV-eGFP) to enable visualization of HIV expression in cells. HIV-eGFP was transfected into HeLa cells with or without FLAG-tagged Nullbasic fused to the mCherry fluorescent reporter protein (Nullbasic-mCherry). We noted strong nuclear accumulation of Nullbasic-mCherry compared to a previous study [18], whereas one exon Tat proteins with basic domain mutation were also shown to predominantly localized in the cytoplasm [20], [23], [24]. Further comparison of Nullbasic localization in HeLa cells showed that Nullbasic fused to FLAG (Fig. S1, rows 3 and 5) or mCherry (Fig. 2A, row 2) had very similar patterns of distribution in the nucleus and cytoplasm. The localization of Rev was determined by indirect immunofluorescence microscopy using an anti-Rev antibody 24 h post-transfection. As expected, Myc epitope-tagged Rev (Myc-Rev) expressed alone was prominent in the nucleolus and the nucleoplasm but to a lesser extent (Fig. 2A, row 1). When Rev was expressed by HIV-eGFP, it was distributed throughout the nucleus (Fig. 2B, row 1). This nuclear distribution of Rev is consistent with its role in nucleocytoplasmic trafficking of viral mRNA [3], [25]. Coexpression of HIV-eGFP and Nullbasic-mCherry resulted in the distinct redistribution of Rev to the nucleoplasm and cytoplasm (Fig. 2B, row2), comparable to the subcellular redistribution of Myc-Rev by Nullbasic-mCherry (Fig. 2A, row2). Although Nullbasic was shown to inhibit Rev-mediated viral mRNA transport leading to limited expression of viral proteins from unspliced or singly spliced viral mRNA [18], these data indicate that Nullbasic-induced redistribution of Rev is independent of other HIV-1 factors under these experimental conditions.

Others have shown that wild type Tat does not interact with Rev within cells [26], [27], [28]. To formally exclude the possibility that Nullbasic may interact with Rev, co-immunoprecipitation reactions were performed using HEK293T cells transfected with plasmids encoding FLAG epitope-tagged Nullbasic (Nullbasic-FLAG) and Myc-Rev. Immunoprecipitation of wild type Tat-FLAG with anti-FLAG beads did not co-immunoprecipitate Myc-Rev (Fig. 2C) but did, as expected, co-immunoprecipitate endogenous CDK9, a member of the pTEF-b protein complex that interacts with the Tat activation domain [29]. Similarly, Nullbasic-FLAG did not show evidence of co-immunoprecipitation of Myc-Rev but did with CDK9 (Fig. 2C). The latter interaction is most likely similar to the interaction between wild type Tat and CDK9, through the activation domain preserved in Nullbasic-FLAG. These experiments imply that a Nullbasic:Rev interaction is not likely to be responsible for the observed mislocalization of Rev.

### CRM1 is Critical for the Nullbasic-mediated Redistribution of Rev from the Nucleolus

In order to investigate the effects of Nullbasic on Rev nucleocytoplasmic transport, we performed the following analyses on Myc-Rev expressed by our Myc-Rev expression plasmid instead of HIV-expressed Rev, since the subcellular localization of Myc-Rev and proviral Rev is similarly altered by Nullbasic.

**Figure 3:**
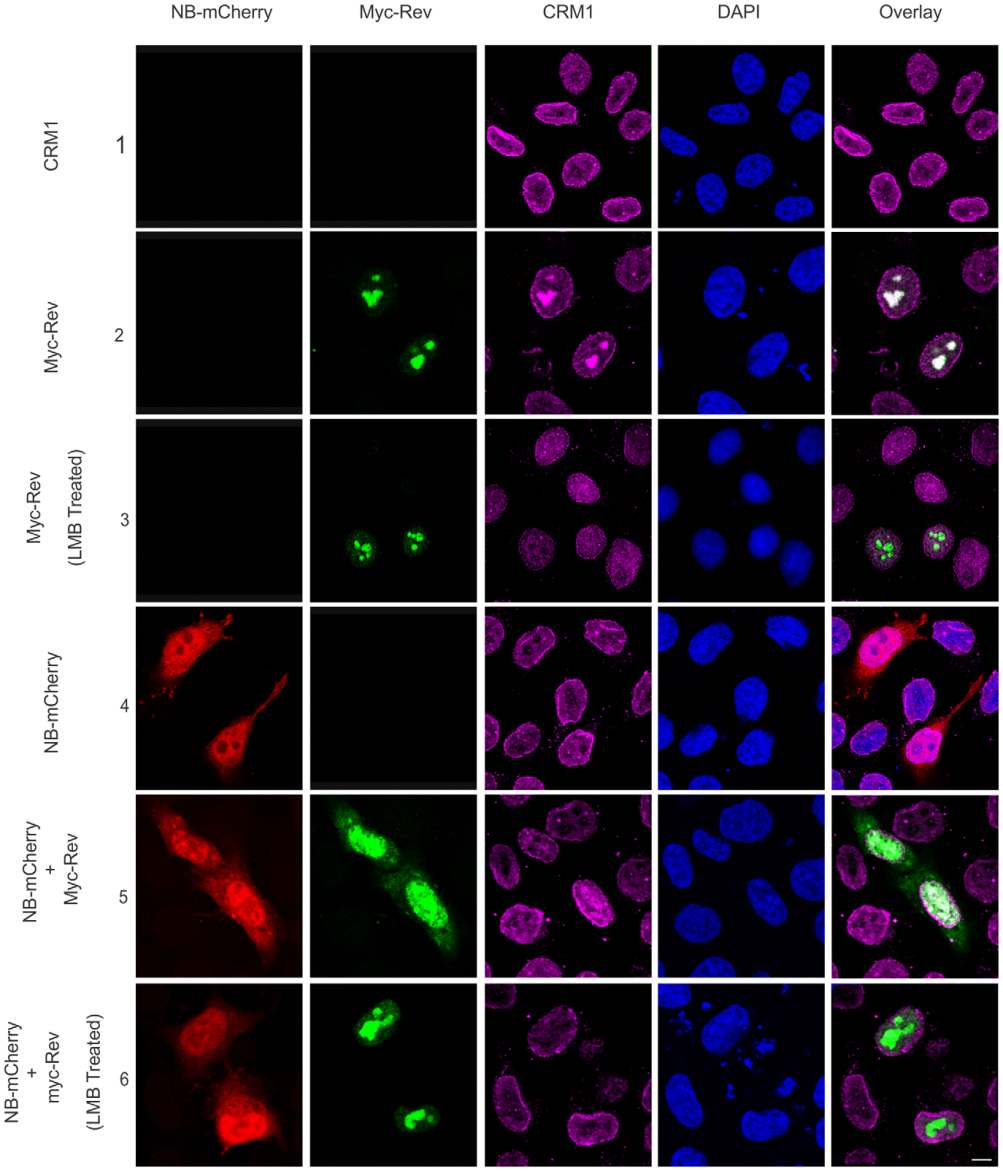
Nullbasic-induced redistribution of Rev is CRM1 dependent. HeLa cells were transfected to express Myc-Rev alone (rows 2 and 3), Nullbasic (NB)-mCherry alone (row 4) or Myc-Rev with NB-mCherry (rows 5 and 6) before being treated with (rows 3 and 6) or without (rows 1, 2, 4 and 6) leptomycin B (LMB) in order to interfere with Myc-Rev and endogenous CRM1 interactions. Cells were fixed and immunostained with anti-Myc (green) and anti-CRM1 (magenta) antibodies and were visualized along with NB-mCherry (red) by fluorescence microscopy. Nuclei were stained with DAPI. The overlay panels show the merge of the Myc-Rev panel with CRM1 panel (rows 1, 2, 3, 5 and 6) and the NB-mCherry with the CRM1 panel (row 4). The figure is representative of five fields each from four independent experiments. The white bar in last panel is equal to 10 µm.

CRM1 can directly bind to the NES sequence in Rev to facilitate its export from the nucleus [3]. We therefore investigated the effect of Nullbasic-mCherry on the association between Myc-Rev and endogenous CRM1 in transfected HeLa cells. In control cells transfected with the parental pcDNA3.1 plasmid, CRM1 was observed widely throughout the nucleus and along the nuclear membrane (Fig. 3, row 1). As previously observed [28], [30], [31], Myc-Rev expression induced CRM1 relocalization and prominent colocalization of Rev and CRM1 in nucleoli (Fig. 3, row 2). This colocalization could be reversed by treating cells with leptomycin B (LMB), a specific CRM1 inhibitor that directly binds to and inactivates CRM1 [32], thereby preventing its interaction with Rev (Fig. 3, row 3). Expression of Nullbasic alone had no apparent effect on CRM1 distribution (Fig. 3, row 4). Colocalization between Rev and CRM1 could also be partially disrupted by the expression of Nullbasic-mCherry, in the presence of which Myc-Rev, but not CRM1, redistributed to the cytoplasm (Fig. 3, row 5). Importantly, treatment of Myc-Rev and Nullbasic-mCherry expressing cells with LMB resulted in the complete restoration of Myc-Rev nucleolar accumulation (Fig. 3, row 6), suggesting that Nullbasic requires functional CRM1 to mislocalize Rev most likely by an indirect interaction.

**Figure 4:**
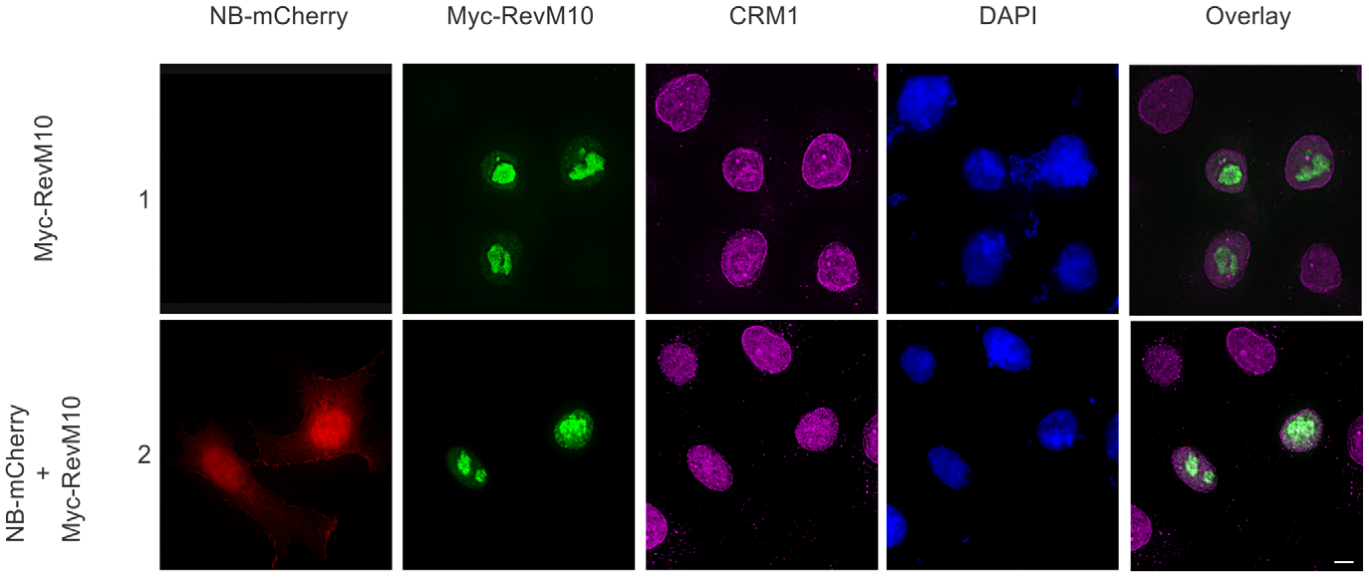
The subcellular localization of the Rev M10 mutant is not altered by Nullbasic. HeLa cells were transfected to express Myc-RevM10 either alone (row 1) or with Nullbasic (NB)-mCherry (row 2). The subcellular localizations of Myc-RevM10 (green), NB-mCherry (red) and endogenous CRM1 (magenta) were visualized by fluorescence microscopy. Nuclei were stained with DAPI. The overlay panels show the merge of the Myc-RevM10 and CRM1 panels. Figures are representative of five fields each from four independent experiments. The white bar in last panel is equal to 10 µm.

To test this hypothesis further, we utilized the Rev mutant RevM10 which contains a non-functional NES and hence is unable to be recognized by CRM1 [33]. As reported for RevM10 [17], [34], Myc-RevM10 (Fig. 4, row 1) was unable to interact and colocalize with CRM1 in the nucleolus. Interestingly, coexpression of Nullbasic-mCherry had no disruptive effect on the nucleolar localization of Myc-RevM10 since, unlike wild type Myc-Rev, no redistribution to the cytoplasm was observed (Fig. 4, row 2). We thus conclude that an intact Rev:CRM1 interaction is required for Nullbasic to relocalize Rev to the cytoplasm.

### Nullbasic Disrupts the Subcellular Localization of Certain Nucleolar Proteins in a Rev-dependent Manner

**Figure 5:**
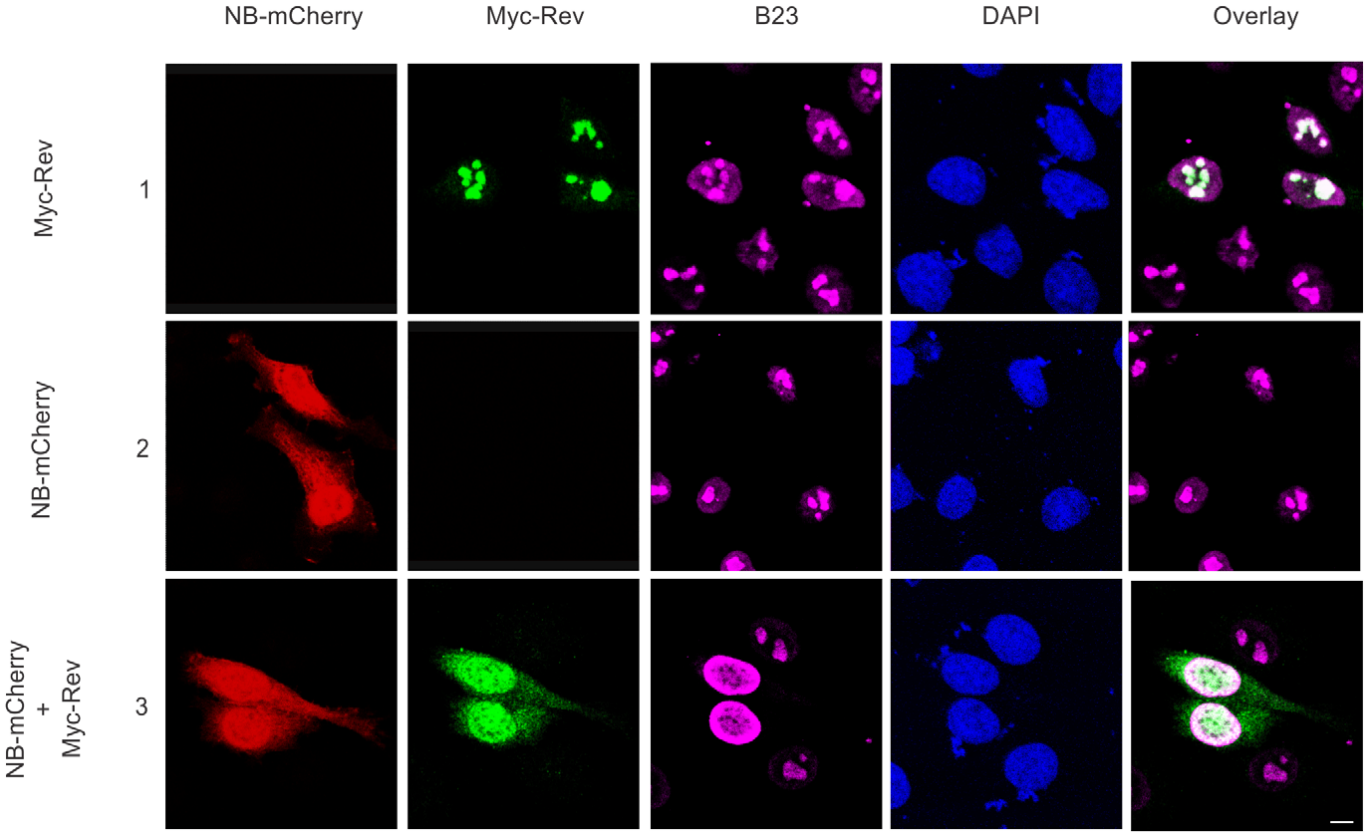
Coexpression of Nullbasic and Rev alters the subcellular localization of B23. HeLa cells were transfected to express Myc-Rev alone (row 1), Nullbasic (NB)-mCherry alone (row 2) or Myc-Rev with NB-mCherry (row 3). Fixed cells were immunostained with anti-Myc (green) and anti-B23 (magenta) antibodies before the subcellular localizations of Myc-Rev, endogenous B23 and NB-mCherry (red) were analyzed by fluorescence microscopy. Nuclei were stained with DAPI. The overlay panels show the merge of the Myc- Rev panel with the B23 panel (rows 1 and 3) and the NB-mCherry panel with the CRM1 panel (row 2). Figures are representative of five fields each from four independent experiments. The white bar in last panel is equal to 10 µm.

B23 is a nucleolar chaperone and shuttling protein that, in HeLa cells, is observed in both the nucleolus and nucleoplasm [35], being associated with nucleolar ribonucleoprotein structures and involved in ribosome biogenesis. B23 is reported to assist Rev and Tat transport into the nucleolus through interactions with their respective basic domains [13], [14], [15], [16], [17], [36]. Mutant Tat proteins lacking a basic domain, like Nullbasic, have been reported not to interact with B23 *in vitro* [14]. As previously observed, Myc-Rev colocalized with B23 in nucleolar structures (Fig. 5, row 1). While expression of Nullbasic-mCherry alone had no apparent effect on B23 nuclear distribution (Fig. 5, row 2), coexpression of Myc-Rev with Nullbasic-mCherry resulted in the strong redistribution of both Myc-Rev and B23 throughout the nucleus, and the observation of Myc-Rev in the cytoplasm (Fig. 5, row 3). Since B23 is known to strongly bind to Rev [13], it is likely that the redistribution of B23 by Nullbasic is Rev-dependent.

**Figure 6:**
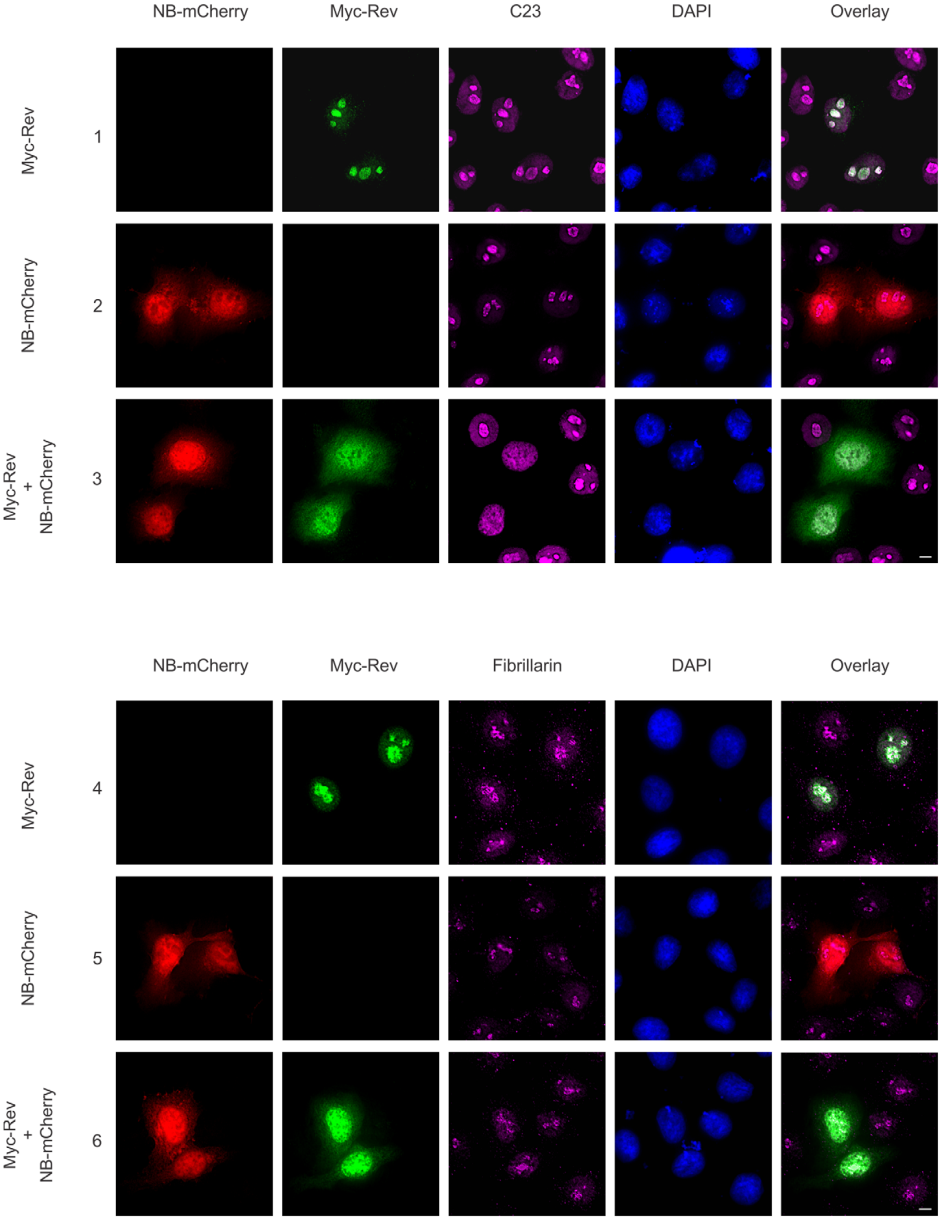
Coexpression of Nullbasic and Rev alters the subcellular localization of C23 but not fibrillarin. HeLa cells were transfected to express Myc-Rev alone (rows 1 and 4), Nullbasic (NB)-mCherry alone (rows 2 and 5) or cotransfected Myc-Rev with NB-mCherry (rows 3 and 6). Fixed cells were immunostained with anti-Myc (green) and either anti-C23 (magenta, upper panels) or anti-fibrillarin (magenta, lower panels) antibodies before the subcellular localizations of Myc-Rev, endogenous C23 or endogenous fibrillarin, and NB-mCherry (red) were analyzed by fluorescence microscopy. Nuclei were stained with DAPI. The overlay panels show the merge of the Myc-Rev panel with either the C23 or fibrillarin panels (rows 1, 3, 4 and 6) and the NB-mCherry panel with either the C23 or fibrillarin panels (rows 2 and 4). All figures are representative of five fields each from four independent experiments. The white bar in last panel is equal to 10 µm.

The data thus far suggest that Nullbasic can alter the localizations of nucleolar proteins in a Rev-dependent manner. To determine if Nullbasic can generally disrupt nucleolar composition, we monitored the localization of C23 and fibrillarin, two nucleolar proteins that have not been reported to interact with Rev. Both C23 and fibrillarin colocalized with Myc-Rev within the nucleoli of transfected cells (Fig. 6, rows 1 and 4, respectively). While expression of Nullbasic-mCherry alone had no apparent effect on the nucleolar localization of either protein (Fig. 6, rows 2 and 5), coexpression of Nullbasic-mCherry with Myc-Rev induced relocalization of C23 but not fibrillarin from the nucleolus to the nucleoplasm, in a manner concomitant with the redistribution of Myc-Rev (Fig. 6, rows 3 and 6, respectively). We therefore conclude that Nullbasic does not cause a generalized mislocalization of nucleolar proteins, but effects the relocalization of only certain, specific proteins (including CRM1, B23 and C23) in a manner dependent on the presence of Rev.

### Nullbasic does not Inhibit Importin β or Transportin 1 Nuclear Import Pathways

**Figure 7:**
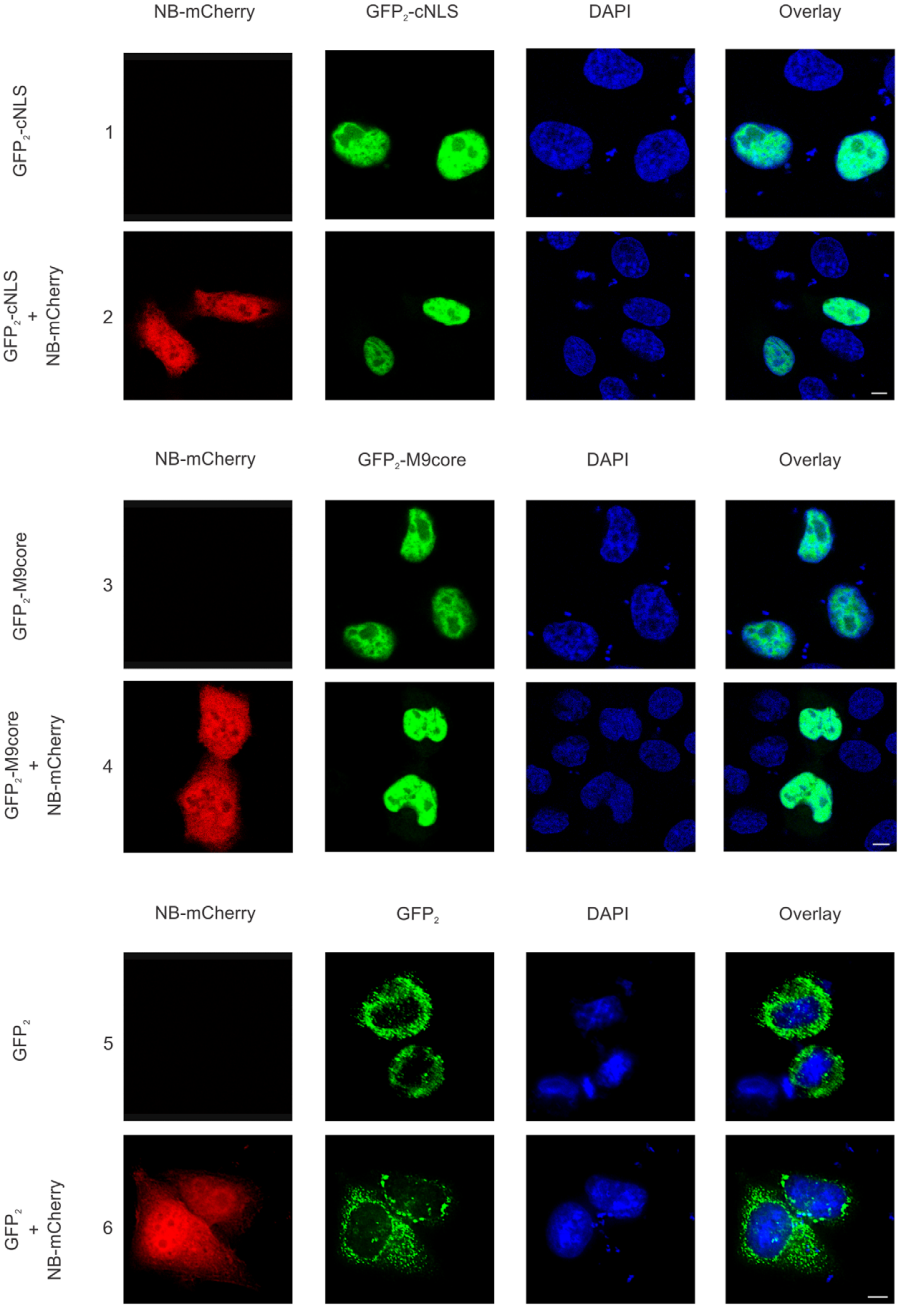
Nullbasic does not affect the nuclear import of importin β and transportin 1 cargoes. HeLa cells were transfected to express the importin β-dependent nuclear import reporter GFP_2_-cNLS, or the transportin 1-dependent nuclear import reporter GFP_2_-M9core, either alone (rows 1 and 3, respectively) or with Nullbasic (NB)-mCherry (rows 2 and 4, respectively). The subcellular localizations of the reporter proteins (green) and NB-mCherry (red) were analyzed in fixed cells by fluorescence microscopy. Nuclei were stained with DAPI. The overlay panels show the merge of the GFP reporter and DAPI panels. Plasmid expressing GFP_2_ was also transfected into cells either alone or with NB-mCherry as a vector control (rows 5 and 6, respectively). Figures are representative of five fields each from four independent experiments. The white bar in last panel is equal to 10 µm.

Five different importin β family members, including importin β, transportin 1, importin 5, importin 7 and importin 9, have been reported to recognize the Rev NLS and facilitate the transport of Rev into the nucleus, with different pathways being used in a cell line-dependent manner [22], [37], [38], [39]. In HeLa cells, for example, transportin 1 is the dominant Rev nuclear import pathway [22], [40], while the importin β pathway is favored in HEK293T cells [40]. To eliminate the possibility that Nullbasic-mediated blockade of a nuclear import pathway leads to the observed cytoplasmic accumulation of Rev, two NLS-containing reporters, GFP_2_-cNLS and GFP_2_-M9core, which mimic cargo for nuclear import by importin β and transportin 1 respectively, were expressed individually or along with Nullbasic-mCherry in HeLa cells. As shown in figure 7, there was no change in the nuclear accumulation of either reporter in the presence of Nullbasic-mCherry (Fig. 7, rows 2 and 4) compared to in its absence (Fig. 7, rows 1 and 3). Nullbasic-mCherry did not affect localization of GFP_2_ lacking a nuclear import signal (Fig. 7, rows 5 and 6). Similar experiments indicated that coexpression of Myc-Rev with Nullbasic-mCherry likewise had no effect on the nuclear localizations of the respective reporters (data not shown). While we cannot exclude interference of other pathways, we conclude that the accumulation of Rev in the cytoplasm induced by Nullbasic is not caused by a general inhibition of the importin β and transportin 1 nuclear import pathways.

## Discussion

This study aimed to elucidate a possible mechanism by which the two-exon anti-viral acting Tat mutant, Nullbasic, perturbs Rev subcellular localization through the active mislocalization of a set of cellular proteins that control Rev trafficking. Nucleocytoplasmic shuttling of Rev is critical for HIV-1 infectivity since viral protein synthesis, and subsequent virion assembly, require the coordinated transport of incompletely spliced viral mRNAs from the nucleus. Nullbasic’s action in downregulating Rev’s mRNA transport function by mechanisms that include the substantial mislocalization of Rev from nucleolus to nucleoplasm and cytoplasm (see Fig. 2) [18] is therefore of great importance. Immunoprecipitation of either Nullbasic-FLAG or Tat-FLAG from lysates of cells co-expressing Myc-Rev revealed no co-immunoprecipitation of Myc-Rev (Fig. 2C), supporting the idea that Nullbasic does not interact with Rev. Furthermore, fluorescence resonance energy transfer analyses between a Tat-GFP fusion protein and a Rev-BFP fusion failed to detect any interaction in living cells [26], [27], a result which we reason will likewise apply to Nullbasic and Rev. It is thus reasonable to conclude that Nullbasic interferes with Rev nucleolar localization by an indirect mechanism.

The fact that Nullbasic strongly disrupts the nucleolar localization of Rev in an indirect manner suggests that Nullbasic may interfere with a cellular factor important for Rev nucleolar accumulation. The nucleolus is a complex structure where several thousands of different cellular proteins have been identified [41]. Among these, CRM1 is a Rev binding protein that regulates Rev trafficking throughout the nucleus. CRM1 is a cellular receptor that recognizes and binds to the leucine-rich NES domain of Rev, an interaction which facilitates the egress of Rev/viral mRNA complexes through nuclear pores [3], [4], [42] and is readily visualized in cells as a colocalization in the nucleolus (Fig. 3, row 2). Our evidence strongly indicates that CRM1 is a mediator through which Nullbasic induces Rev redistribution, since disrupting the interaction between CRM1 and Rev mitigates the effect of Nullbasic. This was evident through the use of LMB (Fig. 3), a specific inhibitor of CRM1, and the RevM10 mutant, whose altered NES is no longer recognized or bound by CRM1 (Fig. 4). In both cases, Rev retained a nucleolar localization despite high levels of Nullbasic expression. As sole expression of Nullbasic does not appear to directly affect CRM1 localization (Fig. 3, row 1 compared to row 4), the precise mechanism by which CRM1 mediates Nullbasic-induced redistribution remains to be elucidated.

B23, C23 and fibrillarin are among the most abundant proteins in the nucleolus [43]. B23 is a nucleolar shuttling factor that recognizes and binds to the NoLS of Rev [13], Tat [44] and C23 [45]. While primarily located in the nucleolus, B23 can display a nucleocytoplasmic distribution as a consequence of its shuttling function [46]. Nullbasic-mediated redistribution of Rev in this study was accompanied by the mislocalizations of B23 (Fig. 5) and C23 (Fig. 6), but not fibrillarin (Fig. 6). It is likely that the mislocalization of B23 in the presence of redistributed Rev is a consequence of their strong interaction [13] rather than through an interaction between Nullbasic and B23 because, while Tat has been reported to interact with B23 through its basic domain [44], the same domain in not present in Nullbasic [18]. Due to the lack of evidence of a direct interaction between C23 and Rev, the mislocalization of C23 may be a result of its interaction with mislocalized B23 [45]. The observed mislocalizations, however, were largely confined to the nucleoplasm even when Rev was substantially redistributed into the cytoplasm.

It is important to note that Nullbasic did not affect the localizations of CRM1, B23 and C23 in the absence of Rev (Figs. 3, 4, 5 and 6). That Nullbasic did not affect the nucleolar distribution of fibrillarin (Fig. 6), either in the presence or absence of Rev, indicates that Nullbasic is not inducing a generalized deformation or reconfiguration of cellular nucleoli. Further, the retention of the importin β and transportin 1 nuclear import reporters in the nucleus in the presence of Nullbasic demonstrates that Nullbasic does not effect a general perturbation of these trafficking pathways (Fig. 7). It is evident, therefore, that Nullbasic induces the redistribution of CRM1, B23 and C23 only in the presence of Rev.

Considering all of the available data, we propose that Rev induces the formation of a nuclear or nucleolar complex to reconcile why Nullbasic redistributes only certain nucleolar proteins in a Rev-dependent manner. This Rev containing complex would be enriched for CRM1 and colocalized with B23 and C23 in order to facilitate the proper processing and nuclear export of HIV-1 unspliced and singly-spliced transcripts [27], [47], [48]. Nullbasic may destabilize the complex that includes Rev by binding to an unidentified factor that is important for its formation, resulting in the redistribution of Rev and concomitant redistributions of B23 and C23 throughout the nucleoplasm. The liberated Rev:CRM1 complexes would freely traffic out of the nucleus, resulting in the observed redistribution of Rev into the cytoplasm, and which would be counteracted by LMB treatment or the RevM10 mutation.

Similar nucleolar hijacking and modification have been described for other viruses. The replication of certain DNA and RNA viruses (such as adenovirus, herpes simplex virus, hepatitis B virus, HIV-1, hepatitis C virus, Japanese encephalitis virus, Hendra virus, Nipah virus, SARS coronavirus and dengue virus) require the trafficking of viral proteins through the nucleolus [42]. In some instances, for example during herpes simplex virus and adenovirus infections, this results in the dramatic mislocalization of C23 and B23 [42], [49]. Given such evidence from other viruses, it is entirely consistent that Rev may similarly manipulate nucleoli factors in HIV-1 infected cells.

Identification of Nullbasic interacting factors that are required for Rev nucleolar accumulation may provide insights into the design of potent yet specific antiretroviral inhibitors. Overall, our data provide evidence that Nullbasic appears to interact with and disrupt specific components of a Rev trafficking complex important for Rev’s nucleocytoplasmic trafficking and nucleolar accumulation, resulting in CRM1-mediated Rev redistribution. Mutant HIV-1 proteins such as Nullbasic thus not only provide insights into the functions of their wild type counterparts, but may also reveal novel drug targets in previously unrecognized host/pathogen interactions.

### Conclusions

CRM1-dependent subcellular redistribution of Rev from the nucleolus by Nullbasic is not through general perturbation of either nuclear import or export. Rather, our data suggest that Nullbasic appears to interact with and disrupt specific components of a Rev trafficking complex required for Rev nucleocytoplasmic shuttling and, in particular, nucleolar accumulation.

## Supporting Information

Figure S1 **Nullbasic-FLAG alters subcellular localization of Rev protein.** HeLa cells were transfected to express Myc-Rev alone (row 1), Tat-FLAG alone (row 2), Nullbasic (NB)-FLAG alone (row 3), Myc-Rev with Tat-FLAG (row 4) or Myc-Rev with NB-FLAG (rows 5). Fixed cells were immunostained with anti-Myc (green) and anti-FLAG (red) antibodies and visualized by fluorescence microscopy. Nuclei were stained with DAPI. The overlay panels show the merge of the Myc-Rev with Tat-FLAG or NB-FLAG panels. The figure is representative of five fields each from four independent experiments. The white bar in last panel is equal to 10 µm.
(TIF)
