# Supplementary figures and images for: Nullbasic, a Potent Anti-HIV Tat Mutant, Induces CRM1-Dependent Disruption of HIV Rev Trafficking
Source: PLoS One. 2012 Dec 10;7(12):e51466. doi: 10.1371/journal.pone.0051466 (PMC3519632; doi:10.1371/journal.pone.0051466)

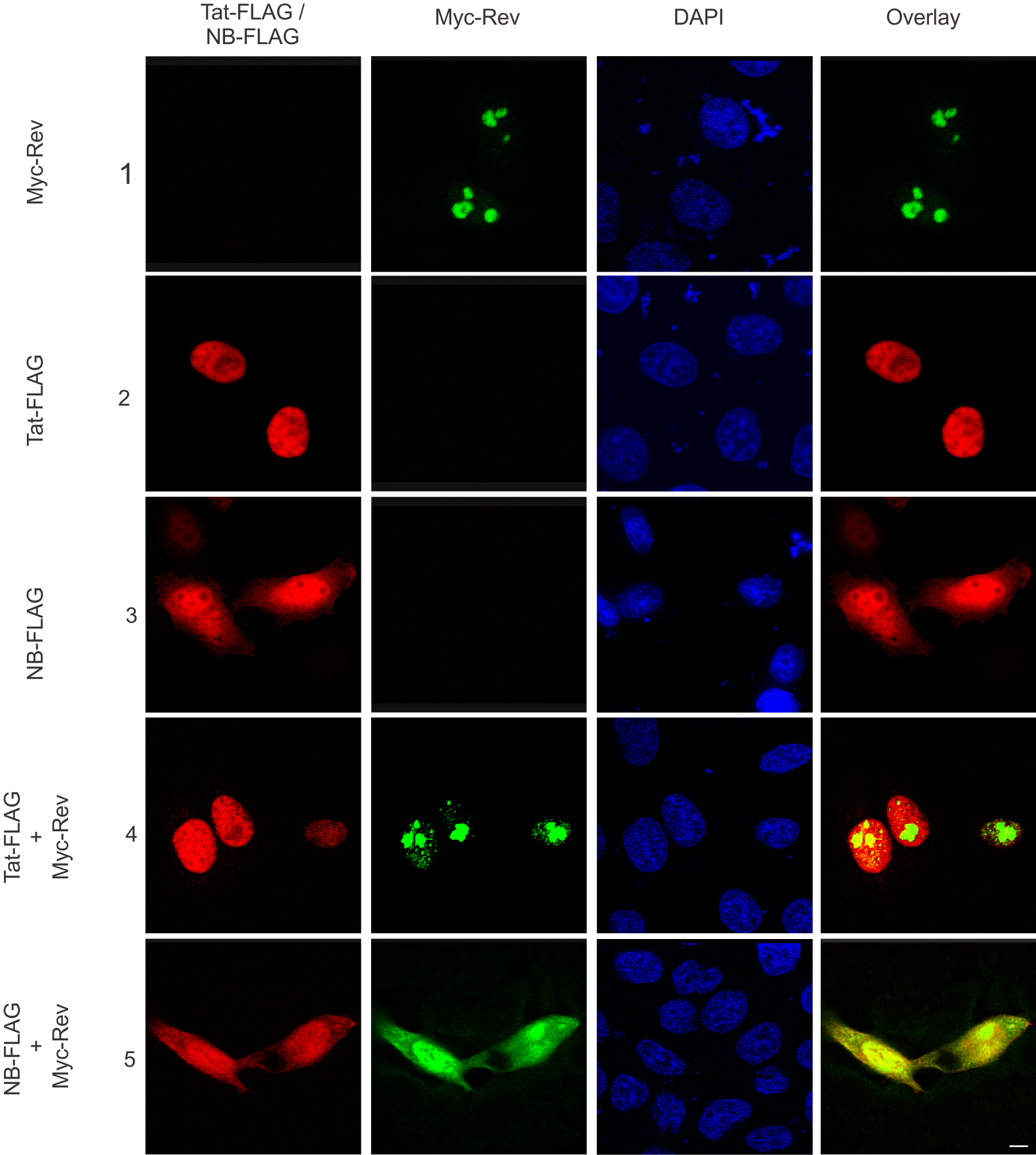

Supplement: Figure S1 — Nullbasic-FLAG alters subcellular localization of Rev protein. HeLa cells were transfected to express Myc-Rev alone (row 1), Tat-FLAG alone (row 2), Nullbasic (NB)-FLAG alone (row 3), Myc-Rev with Tat-FLAG (row 4) or Myc-Rev with NB-FLAG (rows 5). Fixed cells were immunostained with anti-Myc (green) and anti-FLAG (red) antibodies and visualized by fluorescence microscopy. Nuclei were stained with DAPI. The overlay panels show the merge of the Myc-Rev with Tat-FLAG or NB-FLAG panels. The figure is representative of five fields each from four independent experiments. The white bar in last panel is equal to 10 µm. (TIF) [file pone.0051466.s001.tif]
